# Assessment of self‐rated health 5 years after Roux‐en‐Y gastric bypass for severe obesity

**DOI:** 10.1002/bjs5.50223

**Published:** 2019-09-30

**Authors:** J. Sandvik, T. Hole, C. A. Klöckner, B. E. Kulseng, A. Wibe

**Affiliations:** ^1^ Clinic of Medicine and Rehabilitation Møre and Romsdal Hospital Trust Aalesund Norway; ^2^ Centre for Obesity, Department of Surgery St Olav Hospital, Trondheim University Hospital Trondheim Norway; ^3^ Department of Surgery St Olav Hospital, Trondheim University Hospital Trondheim Norway; ^4^ Obesity Research Group, Department of Clinical and Molecular Medicine Norwegian University of Science and Technology Trondheim Norway; ^5^ Faculty of Medicine and Health Sciences Norwegian University of Science and Technology Trondheim Norway; ^6^ Department of Clinical and Molecular Medicine Norwegian University of Science and Technology Trondheim Norway; ^7^ Department of Psychology Norwegian University of Science and Technology Trondheim Norway

## Abstract

**Background:**

Patients' perceptions of health change after bariatric surgery are complex. The aim of this study was to explore whether self‐rated health (SRH), a widely used tool in public health research, might be relevant as an outcome measure after Roux‐en‐Y gastric bypass (RYGB) for severe obesity.

**Methods:**

This was a single‐centre retrospective study of a local quality registry. SRH score was registered at baseline and 5 years after RYGB. SRH, one of the 36 items in the quality‐of‐life Short Form 36 (SF‐36®) questionnaire, is the answer to this single question: ‘In general, would you say your health is excellent (1), very good (2), good (3), fair (4) or poor (5)?’ Change in SRH was analysed in relation to change in weight, co‐morbidities and quality of life after 5 years.

**Results:**

Of a total of 359 patients who underwent RYGB between September 2006 and February 2011, 233 (64·9 per cent) reported on SRH before and 5 years after surgery. Of these, 180 (77·3 per cent) were women, and the mean(s.d.) age was 40(9) years. Some 154 patients (66·1 per cent) reported an improvement in SRH, 60 (25·8 per cent) had no change, and SRH decreased in 19 patients (8·2 per cent). SRH in improvers was related to better scores in all SF‐36® domains, whereas SRH in non‐improvers was related to unchanged or worsened scores in all SF‐36® domains except physical function.

**Conclusion:**

Two‐thirds of patients reported improved SRH 5 years after RYGB for severe obesity. In view of its simplicity, SRH may be an easy‐to‐use outcome measure in bariatric surgery.

## Graphical abstract

A single question on self‐rated health (SRH) could provide an easy‐to‐use outcome measure in bariatric surgery. Two‐thirds of patients who had Roux‐en‐Y gastric bypass for severe obesity reported improved SRH after 5 years.

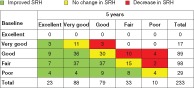

Self‐rated health ‐ One question to replace a questionnaire?

## Abstract (es)

**Antecedentes:**

Las percepciones de los pacientes del cambio de su salud tras la cirugía bariátrica son complejas. El objetivo de este estudio fue examinar si la autovaloración de la salud (*self‐rated health*, SRH), una herramienta ampliamente utilizada en investigación en salud pública, podría ser relevante como medida de resultado después del bypass gástrico en Y de Roux (*Roux‐en‐Y gastric bypass*, RYGB) para la obesidad severa.

**Métodos:**

Se llevó a cabo un estudio retrospectivo de un registro de calidad local de un único centro. La SRH se recogió en situación basal y a los 5 años tras el RYGB. SRH es una de las 36 preguntas del cuestionado de calidad de vida SF‐36 (forma corta) y consiste en la respuesta a esta única pregunta: “En general, diría que su salud es excelente (1), muy buena (2), buena ( 3), razonable (4) o pobre (5)?”.

**Resultados:**

De un total de 359 pacientes sometidos a un RYGB en el periodo entre septiembre de 2006 y febrero de 2011, 233 (65%) informaron del SRH antes y a los 5 años de la cirugía. De ellos, 180 (77,3%) eran mujeres y la edad media era de 40 (DE 9) años. La SRH mejoró en 154 (66,1%) pacientes, no experimento cambios en 60 (25,8%) y disminuyó en 19 (8,2%). En tanto que la mejoría de la SRH se relacionó con mejores puntuaciones en todos los dominios de cuestionario SF‐36, la no mejoría se relacionó con puntuaciones iguales o peores en todos los dominios del SF‐36, a excepción de la función física.

**Conclusión:**

Dos tercios de los pacientes reportaron mejoría de la SRH a los 5 años tras un RYGB por obesidad grave, y debido a su simplicidad, la SRH podría constituir una medida de resultado, de fácil uso, en cirugía bariátrica.

## Introduction

The patient's experience of improvement in general health is the ultimate goal for all medical treatment. The perception of health has several aspects, and the WHO defines health as ‘a state of complete physical, mental and social well‐being and not merely the absence of disease or infirmity’^1^. As severe obesity and bariatric surgery affect all of these aspects, and the most important factors motivating patients to consider seeking bariatric surgery are physical health and longevity^2^, ^3^, measuring weight change alone seems insufficient to evaluate the global effect of this treatment.

Generic as well as disease‐specific tools have been used to evaluate change in quality of life (QoL) after bariatric surgery^4^. Generally, these measures are comprehensive and time‐consuming, and more useful in research than in clinical settings. An association between improvement in QoL and objective improvement in health has not been documented.

Self‐rated health (SRH) is a person's subjective evaluation of their general health, expressed as the answer to the question: ‘In general, would you say your health is excellent, very good, good, fair, or poor?’^5^, ^6^, ^7^, ^8^. In public health surveys and sociological research, SRH has been the most widely used health indicator since the 1950s^9^. Owing to its simplicity, SRH has proved to be a more valid and powerful predictor of morbidity, mortality and healthcare use than more comprehensive self‐reporting instruments and objective biometric measures predicting future health^10^, ^11^, ^12^. Interpreted as a spontaneous subjective assessment of a person's health status, SRH is regarded as the most precise measure of actual experienced health^13^. Public health surveys from different countries and social contexts have documented a relationship between SRH and genetic factors, inflammation and allostatic load, indicating a connection between SRH and biological processes^14^, ^15^, ^16^, ^17^, ^18^, ^19^.

People suffering from severe obesity report lower SRH than the non‐obese, even in the absence of chronic disease^20^. However, despite many advantages, bariatric surgery also has some adverse effects^21^, ^22^, ^23^, ^24^. As a general measure of perceived health, SRH might express the sum of positive and negative aspects of life as experienced by patients in the aftermath of the surgical procedure^25^. Nevertheless, there appear to be no publications on change in SRH after bariatric surgery.

The present study explored whether SRH, a patient‐reported, simple and robust instrument from public health research, is applicable as an outcome measure in bariatric surgery. The primary aim of the study was to evaluate change in SRH from before to 5 years after Roux‐en‐Y gastric bypass (RYGB) for severe obesity. The secondary aim was to explore the relationship between change in SRH to weight loss, co‐morbidity and change in QoL.

## Methods

This study is a retrospective analysis of patients who had RYGB at Aalesund Hospital, a public, non‐academic, secondary referral centre covering a population of 260 000 in Norway. The indication for RYGB was a BMI above 40 kg/m^2^ or a BMI above 35 kg/m^2^ with obesity‐related co‐morbidity in an adult population. The SRH response was collected as part of the Short Form 36 (SF‐36®; QualityMetric, Lincoln, Rhode Island, USA) questionnaire about 1 month before the operation, at the end of a preoperative education day^26^. Answers had no influence on the decision regarding whether the patient would have the operation or not.

SRH is the first question of the SF‐36®, and the version used in this study was the Norwegian translation of the question and alternative answers: ‘In general, would you say your health is (1) excellent, (2) very good, (3) good, (4) fair or (5) poor?’.

Data for all patients who had RYGB at Aalesund Hospital between September 2006 and February 2011 were collected prospectively in a local quality registry, and data from routine visits at 6 weeks and 6, 12, 18, 24, 36, 48 and 60 months after surgery were updated to January 2018. Participation in postoperative support groups, adverse events, plastic surgery and new symptoms related to the bariatric procedure were also registered.

The difference between baseline SRH scores and scores at 5 years was calculated, and the change in SRH was categorized as improvement, no change, or a decrease.

Weight development from baseline through 5 years was reported by standard measures: percentage excess weight loss (%EWL), percentage excess BMI loss (%EBMIL), percentage total weight loss (%TWL) and change in BMI^27^. Weight regain, from nadir weight occurring between 1 and 2 years after surgery to 5 years, was reported as change in BMI and percentage of maximum weight loss^28^.

The study was approved by the Regional Ethics Committee (REK 2016/331) and by the local Data Protection Officer.

### Statistical analysis

Categorical variables are given as proportions. All but one of the continuous variables (SF‐36® physical function sum‐score) were normally distributed and are given as mean(s.d.) values. SRH acts as a categorical as well as a continuous variable. Pearson's χ^2^ test was performed for comparison of categorical variables, and independent and paired *t* tests were performed for comparison of continuous variables. Multiple logistic regression analysis was used to explore whether baseline variables could predict changes in SRH. *P* < 0·050 was considered statistically significant for all analyses. All analyses were performed using IBM SPSS® version 23 (IBM, Armonk, New York, USA).

## Results

A total of 359 patients underwent laparoscopic RYGB as a primary bariatric procedure between September 2006 and February 2011. At baseline, 339 patients completed the SF‐36® questionnaire. After the operation, 322 patients (89·7 per cent) attended the 5‐year follow‐up visit, of whom 242 completed an identical questionnaire. There were complete baseline and postoperative SF‐36® data, as well as clinical information on weight, co‐morbidity, complications and blood test results, for 233 patients, representing 64·9 per cent of patients undergoing RYGB at this hospital in the study period.

Of the 233 patients who formed the study cohort, 180 were women (77·3 per cent) and 53 were men (22·7 per cent). All participants were Norwegian/Caucasian by ethnicity. At baseline, their mean(s.d.) age was 40(9) years and BMI was 43·4(5) kg/m^2^. Nadir BMI was 27·7(4) kg/m^2^, and BMI at 5 years was 30·9(5) kg/m^2^. Details of co‐morbidity at baseline are shown in *Table* 1.

**Table 1:** Patient characteristics

	SRH improvers (*n* = 154)	SRH non‐improvers (*n* = 79)	*P* †
**Age (years)** *	39·9(9·0)	39·5(9·1)	0·711
**Sex ratio (F** : **M)**	115 : 39	65 : 14	0·190‡
**BMI (kg/m** ^**2**^ **)** *			
At baseline	43·2(5·1)	43·8(4·7)	0·374
Nadir	27·7(3·9)	27·7(4·0)	0·993
At 5 years	30·5(4·9)	31·8(5·2)	0·057
**Weight (kg)** *			
At baseline	124·9(18·7)	126·1(19·6)	0·634
Nadir	79·6(13·8)	79·3(15·8)	0·885
At 5 years	88·3(17·0)	92·0(20·7)	0·138
**BMI ≤ 35 kg/m** ^**2**^			
At 1 year	140 (90·9)	69 (87)	0·396‡
At 5 years	121 (78·6)	58 (73)	0·377‡
**%EWL > 50% at 5 years**	124 (80·5)	58 (73)	0·215‡
**%EWL at 5 years** *	71·0(23·9)	64·3(23·9)	0·044
**%EBMIL at 5 years** *	71·6(24·0)	65·0(24·5)	0·049
**%TWL at 5 years** *	29·2(9·6)	27·2(10·3)	0·132
**Change in BMI at 5 years (kg/m** ^**2**^ **)** *	12·7(4·9)	12·0(5·0)	0·295
**Change in BMI from nadir to 5 years (kg/m** ^**2**^ **)** *	2·8(2·5)	4·0(2·7)	0·001
**Change in weight from nadir to 5 years (kg)** *	8·7(7·0)	12·0(8·2)	0·002
**Weight regain (% of maximum weight loss)** *	20·0(18·4)	26·6(18·0)	0·010
**Type 2 diabetes mellitus**			
At baseline	28 (18·2)	10 (13)	0·280‡
Remission at 5 years	21	6	0·369
**Hypertension at baseline**	40 (26·0)	18 (23)	0·594‡
**Hyperlipidaemia**	22 (14·3)	7 (9)	0·235‡
**Sleep apnoea at baseline**	40 (26·0)	16 (20)	0·333‡
**Musculoskeletal pain at baseline**	118 (76·6)	61 (77)	0·786‡
**Smoking at baseline**	50 (32·5)	14 (18)	0·019‡
**Abdominal operations after RYGB**	39 (25·3)	18 (23)	0·669‡
**Internal herniation after RYGB**	22 (14·3)	4 (5)	0·034‡
**Cholecystectomy after RYGB**	12 (7·8)	7 (9)	0·778‡
**Abdominal excess skin removal after RYGB**	75 (48·7)	37 (47)	0·787‡
**Births after RYGB**	17 of 115 (14·8)	5 of 65 (8)	0·163‡
**SRH score** *			
At baseline	3·83(0·76)	3·14(0·76)	< 0·001
At 5 years	2·25(0·77)	3·43(0·89)	< 0·001

Values in parentheses are percentages unless indicated otherwise;

* values are mean(s.d.). %EWL, percentage excess weight loss; %EBMIL, percentage excess BMI loss; %TWL, percentage total weight loss; RYGB, Roux‐en‐Y gastric bypass.

† Paired *t* test, except

‡ χ^2^ test.

**Figure 1:**
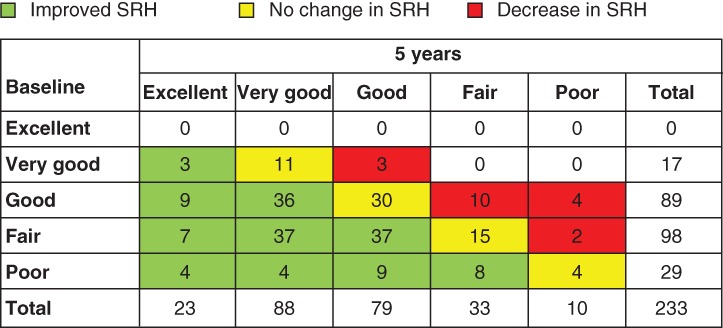
Change in self‐rated health from baseline to 5 years after Roux‐en‐Y gastric bypass for severe obesity SRH, self‐rated health.

**Figure 2:**
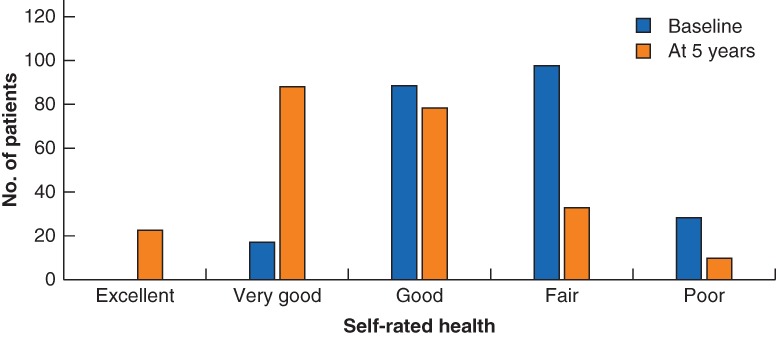
Self‐rated health before and 5 years after Roux‐en‐Y gastric bypass

Mean(s.d.) preoperative SRH was 3·6(0·8), corresponding to a level between ‘good’ and ‘fair’. No patient reported excellent health at baseline, but 17 (7·3 per cent) reported very good SRH, 89 (38·2 per cent) good, 98 (42·1 per cent) fair and 29 (12·4 per cent) poor SRH (*Fig*. 1). At 5 years, mean(s.d.) SRH was 2·7(1·0), corresponding to a level between good and very good; 23 (9·9 per cent) reported excellent, 88 (37·8 per cent) very good, 79 (33·9 per cent) good, 33 (14·2 per cent) fair and ten (4·3 per cent) poor SRH (*Figs* 1 and 2). The proportion reporting fair or poor SRH at baseline was 54·5 per cent (127 of 233), compared with 18·5 per cent (43 of 233) at 5 years.

In terms of individual changes in SRH, 154 patients (66·1 per cent) had a better SRH score at 5 years, 60 (25·8 per cent) had no change, and 19 (8·2 per cent) had a decrease in SRH score (*Fig*. 1). As the number with decreased SRH was low, the variable ‘change in SRH’ was dichotomized to improvers and non‐improvers by merging the no change and decrease categories.

There were no differences between improvers and non‐improvers in terms of age, sex, weight‐related co‐morbidity, or baseline weight and BMI (*Table* 1). At 5 years, mean(s.d.) %EWL was 71·0(23·9) per cent for improvers and 64·3(23·9) per cent for non‐improvers (*P* = 0·044), and %EBMIL was 71·6(24·0) and 65·0(24·5) per cent respectively (*P* = 0·049). There was no significant difference in %TWL (29·2(9·6) per cent for improvers and 27·2(10·3) per cent for non‐improvers; *P* = 0·132), or change in BMI (12·7(4·9) *versus* 12·0(5·0) kg/m^2^ respectively; *P* = 0·295) (*Table* 1).

At 5 years, mean(s.d.) BMI was 30·5(4·9) kg/m^2^ for improvers compared with 31·8(5·2) kg/m^2^ for non‐improvers (*P* = 0·057). Even though there was no significant difference in BMI at 5 years, the improvers had significantly lower weight regain from nadir to 5 years than non‐improvers: 8·7(7·0) *versus* 12·0(8·2) kg respectively (*P* = 0·002), equivalent to a difference in BMI of 1·2 kg/m^2^. Measured as weight regain in percentage of maximum weight loss, from their nadir weight improvers had a weight regain of 20·0(18·4) per cent and non‐improvers 26·6(18·0) per cent (*P* = 0·010) (*Table* 1).

One of the success criteria for bariatric surgery is the achievement of a postoperative BMI of less than 35 kg/m^2^. In total, 179 patients (76·8 per cent) had a BMI of 35 kg/m^2^ or less at 5 years. There was no significant relationship between BMI below or above 35 kg/m^2^ and change in SRH (*P* = 0·377).

Another criterion of success is %EWL of 50 per cent or more, which occurred in 124 (80·5 per cent) of improvers and 58 (73 per cent) of non‐improvers at 5 years (*P* = 0·215). In multiple logistic regression analysis, none of the baseline variables predicted change in SRH (data not shown).

**Figure 3:**
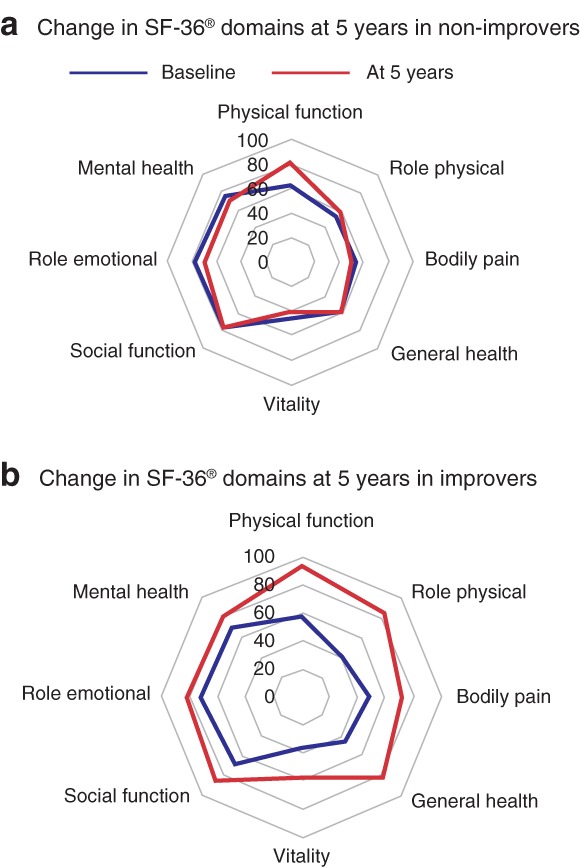
Change in SF‐36® domains among non‐improvers and improvers from baseline to 5 years after Roux‐en‐Y gastric bypass for severe obesity **a** Self‐rated health (SRH) non‐improvers and **b** SRH improvers.

At baseline, improvers had worse sum‐scores than non‐improvers for all SF‐36® domains. However, this difference was not significant for physical function (*P* = 0·065), bodily pain (*P* = 0·149) or role emotional (*P* = 0·137). At 5 years, the opposite relationship was found, as sum‐scores for improvers were significantly better (*P* < 0·050) than those for non‐improvers for all domains. For improvers, sum‐scores at 5 years were better than baseline scores for all eight SF‐36® domains (*P* < 0·005). Non‐improvers had better scores for physical function (*P* < 0·001) and worse scores in mental health (*P* = 0·017) at 5 years compared with the baseline, but no change in the other domains. Details on the relationship between changes in SRH and the eight domains in SF‐36® are given in *Tables* 2 and 3, and *Fig*. 3.

**Table 2:** Change in SF‐36® domain scores among improvers and non‐improvers at baseline and 5 years after Roux‐en‐Y gastric bypass

	Baseline			5 years		
SF‐36® domain	Improvers	Non‐improvers	*P* †	Improvers	Non‐improvers	*P* †
Physical function	57·4(20·1)	62·6(20·7)	0·065	93·4(13·0)	80·9(21·2)	< 0·001*
Role physical	41·0(35·3)	52·2(38·4)	0·026	84·2(31·8)	57·6(40·1)	< 0·001
Bodily pain	48·8(23·5)	54·5(28·9)	0·149	71·2(27·6)	49·7(27·7)	< 0·001
General health	44·5(20·1)	57·5(18·7)	< 0·001	81·8(17·3)	60·0(23·4)	< 0·001
Vitality	36·3(16·82	45·1(18·9)	< 0·001	57·1(21·5)	41·2(23·9)	< 0·001
Social function	67·0(25·9)	76·1(23·3)	0·007	86·4(20·7)	76·1(26·1)	0·003
Role emotional	71·2(36·1)	77·9(33·2)	0·137	82·6(33·3)	69·0(43·6)	0·019
Mental health	69·7(15·4)	75·2(14·7)	0·008	79·2(16·6)	70·6(19·2)	0·001

Values are mean(s.d.).

* At 5 years, the scores for physical function were not normally distributed; the median (i.q.r.) score for improvers was 95 (95–100) and that for non‐improvers 90 (75–95) (*P* < 0·001, Mann–Whitney *U* test).

† Paired *t* test.

**Table 3:** Change in SF‐36® domains from baseline to 5 years after Roux‐en‐Y gastric bypass in improvers and non‐improvers

	Improvers			Non‐improvers		
SF‐36® domain	Baseline	5 years	*P* *	Baseline	5 years	*P* *
Physical function	57·4(20·1)	93·3(13·0)	< 0·001	62·6(20·7)	80·9(21·2)	< 0·001
Role physical	41·0(35·3)	84·2(31·8)	< 0·001	52·2(38·4)	57·6(40·1)	0·314
Bodily pain	48·8(23·6)	71·2(27·6)	< 0·001	54·5 (28·9)	49·7(27·7)	0·158
General health	44·5(20·2)	81·8(17·3)	< 0·001	57·5(18·8)	60·0(23·4)	0·271
Vitality	36·3(16·8)	57·1(21·5)	< 0·001	45·1(18·9)	41·2(23·9)	0·152
Social function	67·0(25·9)	86·4(20·7)	< 0·001	76·1(23·3)	76·1(26·1)	1·000
Role emotional	71·2(36·1)	82·6(33·3)	0·003	77·9(33·2)	69·1(43·6)	0·094
Mental health	69·7(15·4)	79·2(16·6)	< 0·001	75·2(14·7)	70·6(19·2)	0·017

Values are mean(s.d.).

* Paired *t* test.

In terms of co‐morbidity, none of the 19 patients with decreased SRH at 5 years had type 2 diabetes mellitus (T2DM) before surgery. Of the 60 with no change in SRH, ten (17 per cent) had T2DM at baseline and six of these patients did not require medication at 5 years. Of the 154 patients with improved SRH, 28 (18·2 per cent) had T2DM at baseline and 21 did not require medication at 5 years (*Table* 1).

Abdominal surgery for suspected internal herniation was more common among the improvers, but there was no difference between improvers and non‐improvers for cholecystectomy, abdominal excess skin removal or births (*Table* 1).

## Discussion

Before RYGB, patients with severe obesity in the present cohort reported SRH far below that in the general population^29^, but after 5 years their scores were similar, with 81·5 per cent reporting SRH as good, very good or excellent.

QoL scores in SRH improvers were worse than those of non‐improvers at baseline, but they were better at 5 years. Although improved SRH was related to better scores in all SF‐36® domains, non‐improvement was related to unchanged or worsened scores in all domains except physical function. None of the baseline characteristics predicted in which patients perceived health would improve. In a clinical context, these findings may indicate that patients with severe obesity who perceive their health as poor have more to gain from bariatric surgery than patients who perceive their health as good. Moreover, in the long run SRH can be interpreted as the result of the patients' continuous negotiation between the positive and negative effects of the RYGB procedure on all aspects of life.

It terms of the relationship of SRH with weight loss, the study found that the difference between SRH improvers and non‐improvers depended partly on the formula used: %EWL and %EBMIL were better for improvers than for non‐improvers, but %TWL and change in BMI were not different; and the proportion of patients attaining a BMI of 35 kg/m^2^ or less, or %EWL above 50 per cent at 5 years, was similar for improvers and non‐improvers. However, non‐improvers regained 3·3 kg more than improvers from nadir to 5 years after RYGB, a significant difference. Whether this ‘marginal’ weight regain reduced SRH, or whether other health issues led to increased weight among non‐improvers, could not be explored further from the available data.

Considering long‐term outcomes, a meta‐analysis^30^ reported that health‐related QoL improved in the first year after bariatric surgery, declined after 2 years and stabilized at a level below that in the general population at 5 years and, compared with control groups with obesity, improvement in both physical and mental health was reported more than 5 years after surgery^31^. Long‐term observational studies^32^, ^33^, ^34^ of adults with severe obesity have reported that, compared with usual care, bariatric surgery is associated with a reduced rate of cardiovascular events and deaths, but still with a higher mortality rate than in the general population. The sample size in the present study was too small and the observation time too short to explore whether improved SRH after RYGB had an effect on mortality and future morbidity.

The strengths of this study are the close follow‐up and complete registration for many variables from baseline to 5 years after the RYGB, and that patients reported on SRH when they had long‐term experience of the positive and negative effects of the surgery on their general health status. Among the limitations of the study are the small sample size, and that the SF‐36® questionnaire was not given to all patients who attended the 5‐year follow‐up visit. In addition, the study did not consider socioeconomic factors or life events that may have affected SRH at baseline or during follow‐up after the bariatric procedure.

SRH, expressed by the answer to one single question, seems relevant and valid as an outcome measure for bariatric surgery, and in this observational study RYGB for severe obesity resulted in improved SRH in two‐thirds of the patients. Focusing not only on weight, but also on health in general, might reduce the stigma experienced by people with severe obesity considering or undergoing bariatric surgery. The increased knowledge on what to expect from bariatric surgery will be useful for patient education, their choice of treatment, and their view of life after treatment for severe obesity. In clinical use, SRH might replace more comprehensive QoL tools, and SRH scores can be used to identify patients in need of closer follow‐up after surgery.
